# Comorbidity phenotypes and risk of mortality in patients with osteoarthritis in the UK: a latent class analysis

**DOI:** 10.1186/s13075-022-02909-4

**Published:** 2022-10-13

**Authors:** Dawit T. Zemedikun, Helena Lee, Krishnarajah Nirantharakumar, Karim Raza, Joht Singh Chandan, Janet M. Lord, Thomas A. Jackson

**Affiliations:** 1 Institute of Applied Health Research, University of Birmingham, Birmingham, UK; 2 MRC-Versus Arthritis Centre for Musculoskeletal Ageing Research, Institute of Inflammation and Ageing, College of Medical and Dental Sciences, University of Birmingham, Queen Elizabeth Hospital, Edgbaston, Birmingham, B15 2WB UK; 3 Research into Inflammatory Arthritis Centre Versus Arthritis, University of Birmingham, Birmingham, UK; 4 NIHR Birmingham Biomedical Research Centre, University Hospital Birmingham and University of Birmingham, Birmingham, UK; 5 Sandwell and West Birmingham NHS Trust, Birmingham, UK

**Keywords:** Osteoarthritis, Comorbidity, Latent class analysis, Electronic health records, Primary care

## Abstract

**Background:**

Osteoarthritis (OA) is a common chronic condition but its association with other chronic conditions and mortality is largely unknown. This study aimed to use latent class analysis (LCA) of 30 comorbidities in patients with OA and matched controls without OA to identify clusters of comorbidities and examine the associations between the clusters, opioid use, and mortality.

**Methods:**

A matched cohort analysis of patients derived from the IQVIA Medical Research Data (IMRD-UK) database between 2000 and 2019. 418,329 patients with newly diagnosed OA were matched to 243,170 patients without OA to identify comorbidity phenotypes. Further analysis investigated the effect of opioid use on mortality in individuals with OA and their matched controls.

**Results:**

The median (interquartile range (IQR)) number of comorbidities was 2 (1–4) and 1 (0–3) in the OA and control groups respectively. LCA identified six comorbidity phenotypes in individuals with and without OA. Clusters with a high prevalence of comorbidities were characterised by hypertension, circulatory, and metabolic diseases. We identified a comorbidity cluster with the aforementioned comorbidities plus a high prevalence of chronic kidney disease, which was associated with twice the hazard of mortality in hand OA with a hazard ratio (HR) (95% CI) of 2.53 (2.05–3.13) compared to the hazard observed in hip/knee OA subtype 1.33 (1.24–1.42). The impact of opioid use in the first 12 months on hazards of mortality was significantly greater for weak opioids and strong opioids across all groups HR (95% CI) ranging from 1.11 (1.07–11.6) to 1.80 (1.69–1.92)). There was however no evidence of association between NSAID use and altered risk of mortality.

**Conclusion:**

This study identified six comorbidity clusters in individuals with OA and matched controls within this cohort. Opioid use and comorbidity clusters were differentially associated with the risk of mortality. The analyses may help shape the development of future interventions or health services that take into account the impact of these comorbidity clusters.

**Supplementary Information:**

The online version contains supplementary material available at 10.1186/s13075-022-02909-4.

## Introduction

The prevalence of osteoarthritis (OA) increases with advancing age [1]. Currently, there are no disease-modifying agents available for OA, with analgesia, physical therapies, and, where appropriate, surgery, being the mainstays of treatment [2]. An ageing population has resulted in an increase in lifespan but not health span at the same rate; thus, people are living longer but with an increasing number of non-communicable long-term health conditions (multimorbidity) and ill-health [3, 4]. The consequences of this are increased treatment and prescribing burden which is associated with disability and frailty [5, 6].

OA is associated with both multimorbidity and polypharmacy [7, 8]. To date, multimorbidity research has largely focussed on descriptions of disease clustering [9, 10], yet such research has the potential to inform us about disease pathophysiology and underlying mechanisms, disease trajectories within multimorbidity clusters, and implications for disease management. To our knowledge, identification and analysis of multimorbidity clusters in OA have not been described before. This analysis sets out to identify whether there are distinct multimorbidity clusters in patients with OA and if these clusters differ between hand and hip-knee OA subtypes. This is relevant as the pathophysiology of OA may differ with the anatomical site and in particular between load-bearing and non-load-bearing joints [11]. Sex-related differences in the prevalence of both hip/knee OA and hand OA may also be explained by differing underlying pathophysiology [12].

We also aim to identify mortality outcomes associated with comorbidity clusters among OA patients and within subtypes of OA. For example, prescription of opioids, commonly deployed as analgesia in OA, may be associated with increased mortality in patients with OA but these findings may be prone to confounding by indication [13]. Hence, we examine whether opioids are significant contributors towards mortality in OA subtypes adjusting for derived comorbidity clusters.

## Methods

### Study design and data source

This study was a population-based retrospective open cohort design using the IQVIA Medical Research Data (IMRD-UK), previously named ‘The Health Improvement Network (THIN)’. The database consists of UK electronic medical records derived from 830 general practices comprising about 16.6 million patients. IMRD-UK is representative of the UK population in terms of demographic structure and prevalence of common morbidities [14]. Information relating to symptoms, examinations, investigations, and diagnoses are recorded within IMRD-UK as Read codes, a clinical hierarchy coding system [15]. General practices were eligible for inclusion 12 months following their instalment of electronic health records or from the practice’s acceptable mortality recording date [16].

### Study population

Adults aged 18 years or older with a diagnosis of OA (cases) and registered with an eligible general practice were identified during the study period (1 January 2000 to 31 December 2019). The index date for the cases was taken to be the first recorded Read code relating to OA once the patient was eligible to take part in the study. The same index date was assigned to the corresponding control patient matched 1:1 based on age (± 1 year), sex, and general practice. To avoid reverse causality, a lag period was set to ensure follow-up started 1 year after the index date until the exit date defined as the earliest of exit from the IMRD-UK database (transferred practice or died), the last date practice data was collected, or 31 December 2019.

### OA and comorbidity ascertainment

Read codes were used to identify incident diagnoses of OA (Supplementary table S1). Patients with OA were identified as having hand OA, hip/knee OA, and OA ‘with site unspecified’ where Read codes did not provide the information required to stratify by regions affected. We reported on the OA ‘with site unspecified group’, but conducted no further analysis in this subgroup. The presence of comorbidities among patients with OA was defined as having a diagnosis of any of 30 different comorbidities (Supplementary table S2) recorded prior to the diagnosis of OA. For presenting the phenotype analysis, some of the morbidities were combined to enhance the quality of the data, producing a total of 15 disease groups (Supplementary table S3).

### Statistical analysis

Latent class analysis (LCA) was conducted using the 30 comorbidities as observed variables. LCA is a regression-based method that can probabilistically identify homogenous, unobserved subgroups within a heterogeneous group using a set of observed variables [17, 18]. LCA estimates the response probability for each observed disease according to latent class (cluster) membership. It also estimates the proportion of individuals that are expected to belong to each cluster. Further explanation is given as Supplementary methods in Additional file 1. LCA models with two to eight latent classes were assessed and the optimal number of clusters was selected based on model fit criteria (Supplementary table S4) and domain usefulness (clinical interpretability and meaningfulness) [19]. Each patient was assigned to one of the clusters according to their highest computed probability of membership. Each of the comorbidity clusters was labelled according to the prevalence of comorbidities. Patients with no comorbidities were excluded from the analyses.

Kaplan-Meier survival curves were generated for the disease clusters adjusted for age and sex. Cox regression models were used to estimate hazard ratios (HRs) and 95% CIs of mortality for the disease clusters using the lowest comorbidity disease cluster as the reference category. Analyses were adjusted for age, sex, body mass index (BMI), Townsend deprivation score [20], smoking status, ethnicity, and opioid (divided into weak opioids and strong opioids) use and NSAID use in the first 12 months after diagnosis of OA. To ensure that the same patients were being assessed in all analyses, patients with missing values for Townsend, smoking status, and ethnicity were assigned to a separate category for that variable and included in the regression analysis. Due to the lag period introduced, patients with less than 1 year of follow-up were excluded from the mortality analysis.

Subgroup analyses were conducted separately in the hand OA and hip/knee OA groups, and we presented and made descriptive comparisons of the differences in results observed including in the control population. All statistical analyses were performed using Stata 16.1 SE (StataCorp, College Station, TX). The Scientific Review Committee of IQVIA approved the study protocol (SRC Reference Number: 20SRC017) prior to its undertaking.

## Results

### Characteristics of the study population

There were 165,195 men and 253,134 women with a new diagnosis of OA during the study period (Table 1). OA was more common in women than men across all three subgroups while hip/knee OA patients were slightly older (mean (SD): 65 (12.3) years) and had higher BMI (29.2 (5.9)) in comparison to the other two groups. The OA ‘with site unspecified’ group (OA site unknown) is likely to be a mixture of the other two types of OA and also to include other less common types of OA (e.g. ankle, shoulder) and hence is excluded from further analyses. The use of opioids was higher among the hip/knee OA patients in comparison to hand OA. Given the matching process, the socio-demographic characteristics were similar between the overall OA and the control groups.

**Table 1:** Baseline characteristics of the study population by type of OA and their corresponding controls

Description	All OA	Hand OA	Hip/knee OA	OA site unknown	Controls	***p***-value
*N* (%)	418,329 (100)	26,005 (6.2)	137,507 (32.9)	254,817 (60.9)	243,170 (100)	
Follow-up years, mean (SD)	6.1 (4.4)	6.0 (4.2)	6.0 (4.2)	6.3 (4.5)	5.9 (4.4)	<0.001^a^
Comorbidities, median (interquartile range (IQR))	2 (1–4)	2 (1–3)	2 (1–4)	2 (1–4)	1 (0–3)	
Comorbidities, mean (SD)	2.59 (2.1)	2.36 (2.0)	2.64 (2.1)	2.58 (2.1)	1.87 (1.8)	<0.001^a^
Sex (%)						<0.001^b^
Male	165,195 (39.5)	7415 (28.5)	60,376 (43.9)	97,404 (38.2)	99,419 (40.9)	
Female	253,134 (60.5)	18,590 (71.5)	77,131 (56.1)	157,413 (61.8)	143,751 (59.1)	
Age (years), mean (SD)	64.39 (12.5)	61.91 (11.0)	65.40 (12.3)	64.10 (12.8)	60.13 (11.7)	<0.001^a^
BMI (kg/m^2^), mean (SD)	28.45 (5.8)	27.06 (5.3)	29.16 (5.9)	28.21 (5.8)	26.96 (5.4)	<0.001^a^
Smoking status (%)						<0.001^b^
Non-smoker	223,969 (53.5)	14,730 (56.6)	75,173 (54.7)	134,066 (52.6)	130,093 (53.5)	
Smoker	62,545 (15.0)	3626 (13.9)	18,307 (13.3)	40,612 (15.9)	45,383 (18.7)	
Ex-smoker	120,258 (28.8)	7219 (27.8)	40,734 (29.6)	72,305 (28.4)	57,365 (23.6)	
Missing	11,557 (2.8)	430 (1.7)	3293 (2.4)	7834 (3.1)	10,329 (4.3)	
Townsend quintiles (%)						<0.001^b^
1 (least deprived)	95,670 (22.9)	6829 (26.3)	31,454 (22.9)	57,387 (22.5)	63,313 (26.0)	
2	84,521 (20.2)	5805 (22.3)	28,556 (20.8)	50,160 (19.7)	48,214 (19.8)	
3	76,472 (18.3)	4548 (17.5)	25,006 (18.2)	46,918 (18.4)	40,888 (16.8)	
4	62,806 (15.0)	3199 (12.3)	20,598 (15.0)	39,009 (15.3)	30,983 (12.7)	
5 (most deprived)	41,148 (9.8)	1998 (7.7)	12,716 (9.3)	26,434 (10.4)	19,338 (8.0)	
Missing	57,712 (13.8)	3626 (13.9)	19,177 (14.0)	34,909 (13.7)	40,434 (16.6)	
Ethnicity (%)						<0.001^b^
White	187,073 (44.7)	11,872 (45.7)	61,989 (45.1)	113,212 (44.4)	105,374 (43.3)	
Mixed race	1501 (0.4)	108 (0.4)	432 (0.3)	961 (0.4)	536 (0.2)	
Other	635 (0.2)	29 (0.1)	198 (0.1)	408 (0.2)	2045 (0.8)	
Black	3104 (0.7)	68 (0.3)	1047 (0.8)	1989 (0.8)	2282 (0.9)	
South Asian	5713 (1.4)	235 (0.9)	1907 (1.4)	3571 (1.4)	3123 (1.3)	
Missing	220,303 (52.7)	13,693 (52.7)	71,934 (52.3)	134,676 (52.9)	129,810 (53.4)	
NSAID/opioid use (%)						<0.001^b^
None	161,974 (38.7)	13,832 (53.2)	49,505 (36.0)	98,637 (38.7)	77,903 (32.0)	
NSAIDs	113,759 (27.2)	6540 (25.2)	37,060 (27.0)	70,159 (27.5)	89,843 (37.0)	
Weak opiates	132,740 (31.7)	5268 (20.3)	47,490 (34.5)	79,982 (31.4)	71,586 (29.4)	
Strong opiates	9856 (2.4)	365 (1.4)	3452 (2.5)	6039 (2.4)	3838 (1.6)	

^a^ANOVA test and ^b^chi-squared test for differences among the subgroups of OA and controls

### Morbidity differences

The median (IQR) number of comorbidities was 2 (1–4) and 1 (0–3) in the overall OA and control groups respectively. Patients in the subgroups of OA presented with a similar median (IQR) number of comorbidities as the overall OA group. The prevalence of each of the 30 comorbid conditions included is provided in Supplementary table S2. Of these conditions, nine had a prevalence of >10% in the overall OA group and 17 had a prevalence of 1 to 10%, while the remaining four had a prevalence of less than 1%. The proportions of comorbidities were lower among the controls than the overall OA patients.

### Comorbidity clusters

**Fig. 1:**
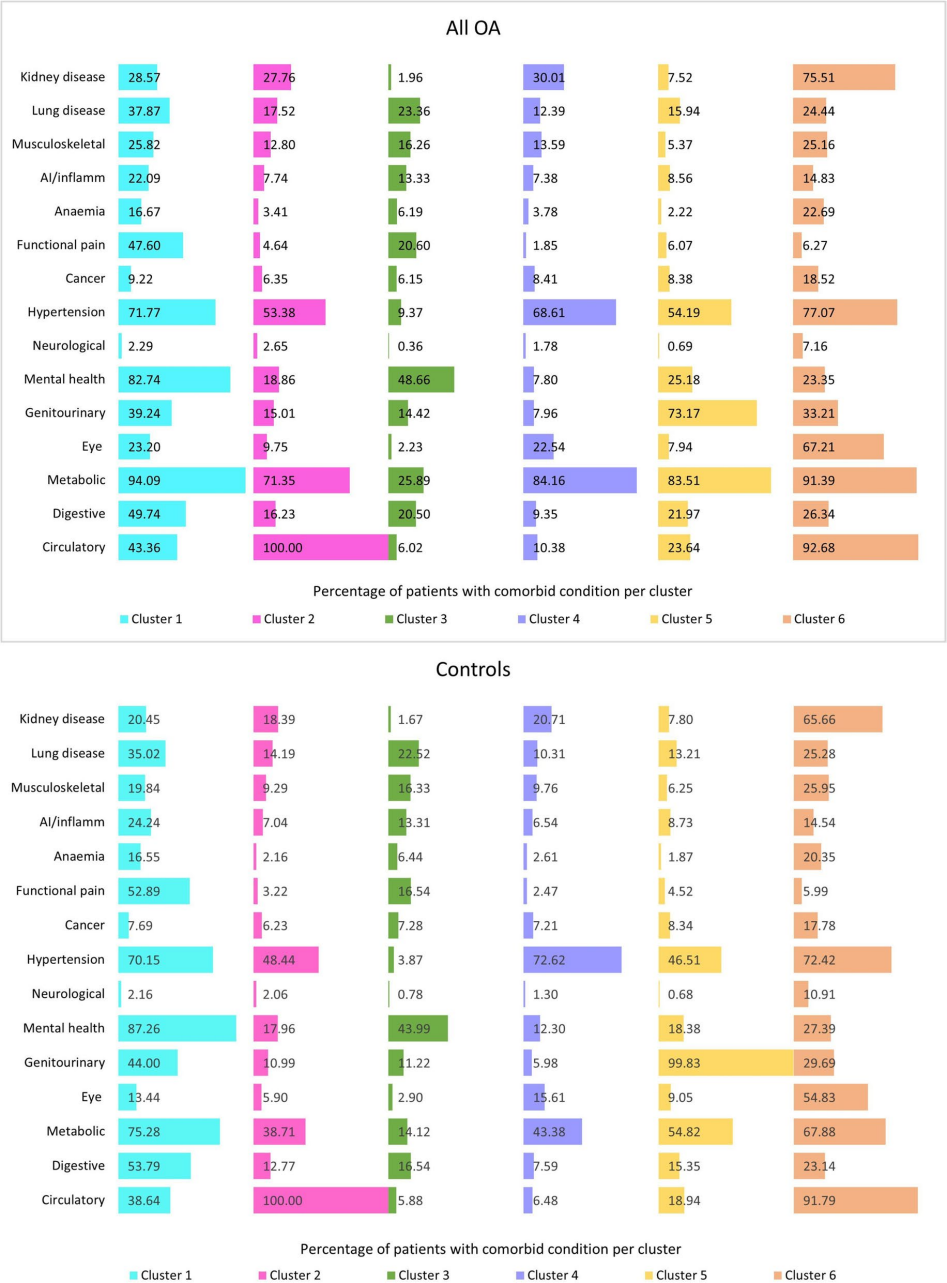
Prevalence of individual (groups of) diseases in the overall OA and matched control population according to comorbidity phenotype at the time of diagnosis of OA

Having considered the model fit information (Supplementary table S4) and clinical interpretability and meaningfulness, a six-class model was identified in each of the LCA conducted. The estimated cumulative percentage (may also be expressed as item-response probabilities) of an individual comorbid disease in each latent class of the overall OA population is shown in Fig. 1. For ease of presentation and reference, some of the diseases were combined into the comorbidity group and, where possible, the clusters are referred to by its lead condition(s) whose cluster-specific prevalence is highest.

Cluster 1 (4.4% of patients) was a high burden cluster characterised by metabolic diseases (94%), mental health conditions (83%), and hypertension (72%). In cluster 2 (11% of patients), all patients had a circulatory disease and 71% also had a metabolic disease. Cluster 3 (40.1% of patients) was the lowest burden cluster (lower prevalence of comorbid conditions) and largely consisted of younger patients. Cluster 4 (31.9% of patients) was also a lower burden cluster in comparison to the other four clusters and was dominated by metabolic diseases (84.2%) and hypertension (68.6%). Patients in cluster 5 (7.5% of patients) had high proportions of coexisting metabolic (83.5%) and genitourinary (73.2%) diseases. Cluster 6 (5.2% of patients) was the highest burden cluster (higher prevalence of comorbid conditions) characterised by circulatory (92.7%), metabolic (91.4%), hypertension (77.1%), and kidney (75.5%) diseases, as well as a high proportion of eye (67.2%) disease. This high burden cluster predominantly consisted of older patients being 75 years of age or over.

Similar clustering patterns were observed in the matched control population, who did not have OA as one of their conditions. There were however some notable differences; metabolic conditions in particular were substantially reduced in the control population in comparison to the cases across all clusters. Another major difference was that cluster 5 in the control population was dominated by genitourinary diseases (99.8%) compared to the cases (73.2%).

**Fig. 2:**
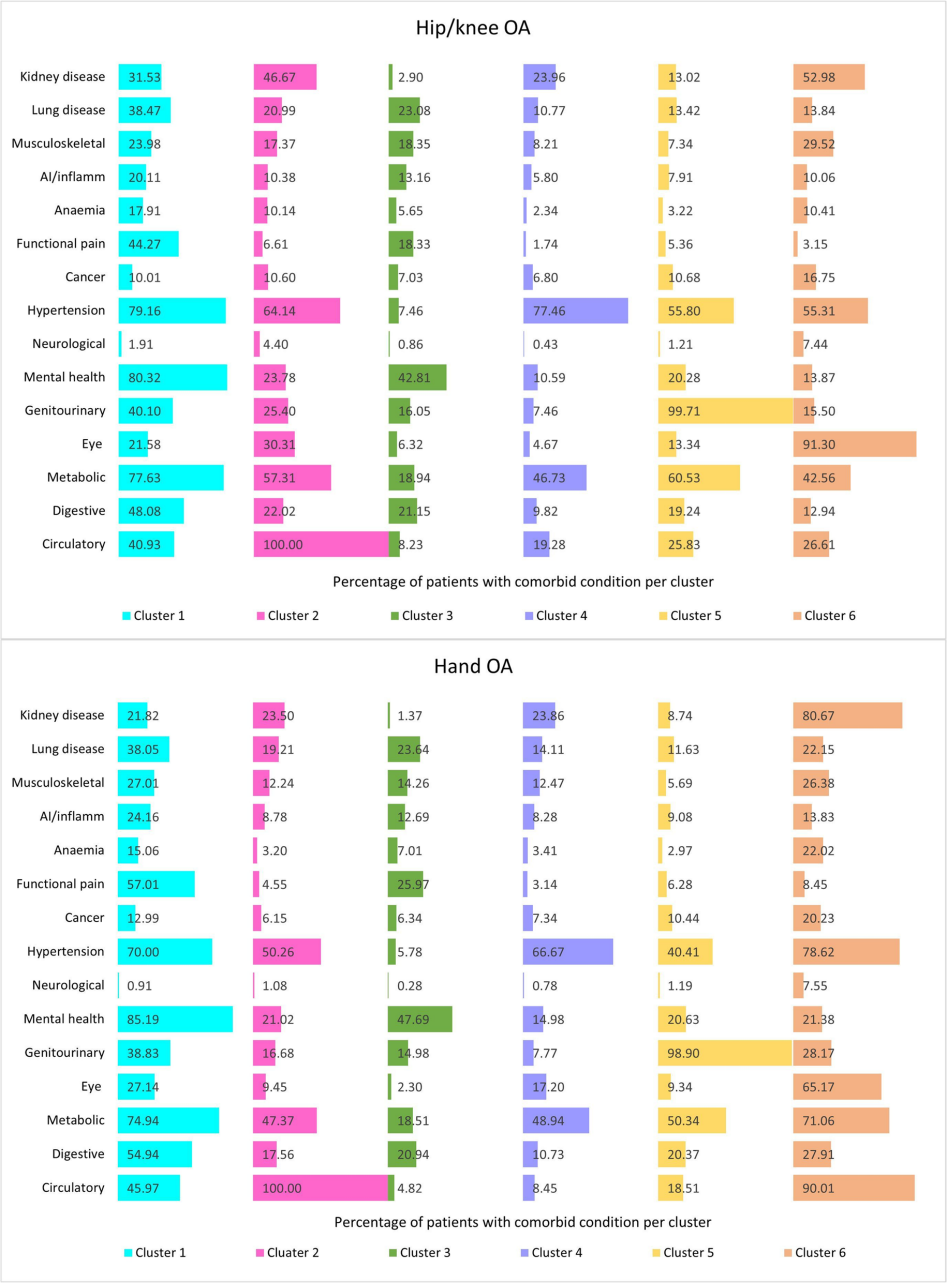
Prevalence of individual (groups of) diseases in the hip/knee OA and hand OA population according to comorbidity phenotype at the time of diagnosis of OA

The results of the subgroup analyses by OA type (Fig. 2) revealed marked differences in the distribution of comorbidities in the clusters (in particular clusters 2, 5, and 6) between the hand OA and hip/knee OA populations. Cluster 6 was a high burden cluster in hand OA but a moderate burden in hip/knee OA, while the compositions were similar. Clusters 2 and 5 also had higher proportions of hypertension and metabolic diseases, as well as kidney disease (cluster 2 only) in the hip/knee in comparison to the hand OA population.

In the overall OA population, cluster 6 predominantly consisted of older patients with 74.5% of that group being 75 years of age or over as opposed to 8.7% in cluster 3. Sex disparities were also significant with cluster 1 being largely female patients (88.7%) and cluster 5 male patients (86.5%). Opioid use was highest in the high burden clusters (clusters 1 and 6) and lowest in the low burden cluster 3. Patterns in the control population followed a similar pattern but the differences were less pronounced particularly sex differences in cluster 5.

### Mortality outcomes and impact of opioid use

**Fig. 3:**
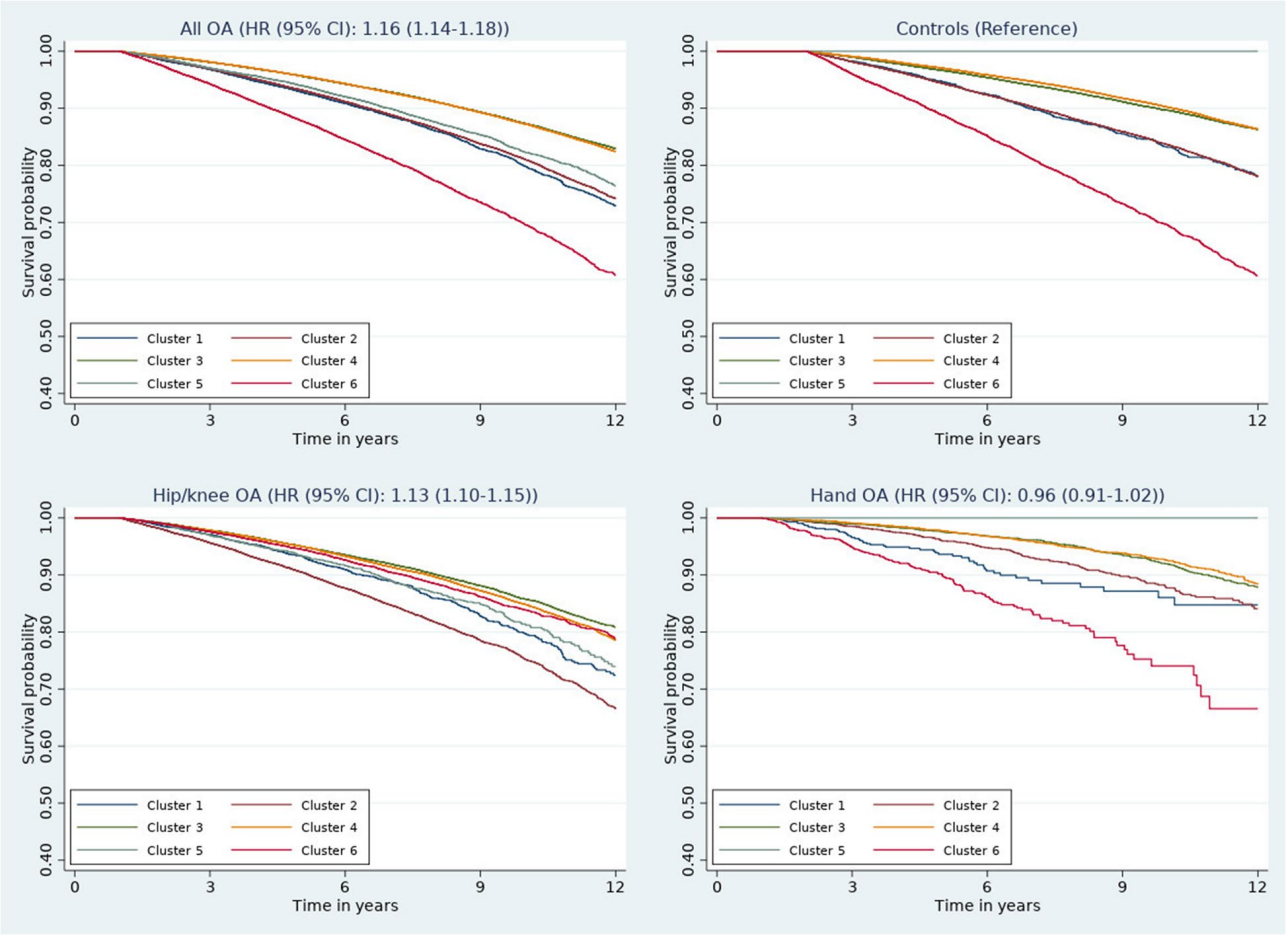
Age- and sex-adjusted Kaplan-Meier survival curves according to comorbidity phenotypes by OA type and in the control population

Survival curves for the disease clusters adjusted for age and sex are shown in Fig. 3. In the overall OA, survival was lowest in cluster 6 (highest burden cluster); moderate in clusters 1, 2, and 5; and highest in clusters 3 and 4. The corresponding hazard ratios (HRs) and 95% confidence intervals (CIs) (Table 2) support this. In addition to age and sex, the model was further adjusted for BMI, deprivation, smoking status, ethnicity, and opioid usage in the first 12 months after diagnosis. Similar patterns were observed in the hand OA and hip/knee OA groups (Fig. 3), but hip/knee patients generally had poorer mortality outcomes in comparison to the hand OA patients. In the matched control population, survival appeared highest in the genitourinary cluster 5 but this was non-significant (Fig. 3).

**Table 2:** Adjusted hazard ratios of mortality by cluster in the different groups. Models were adjusted for age, sex, BMI categories, deprivation quintiles, smoking status, ethnicity, and opioid use in the first 12 months of OA diagnosis

Group	Cluster	aHR	***p***-value	95% CI	
All OA	3	Ref			
	1	1.44	<0.001	1.36	1.51
	2	1.52	<0.001	1.47	1.57
	4	1.16	<0.001	1.13	1.20
	5	1.22	<0.001	1.16	1.28
	6	2.09	<0.001	2.01	2.17
Hand OA	3	Ref			
	1	1.71	<0.001	2.27	2.27
	2	1.55	<0.001	1.30	1.85
	4	1.09	0.243	0.94	1.27
	5	1.14	0.386	0.85	1.53
	6	2.53	<0.001	2.05	3.13
Hip/knee OA	3	Ref			
	1	1.37	<0.001	1.25	1.50
	2	1.71	<0.001	1.62	1.80
	4	1.12	<0.001	1.06	1.17
	5	1.02	0.751	0.91	1.13
	6	1.33	<0.001	1.24	1.42
Controls	3	Ref			
	1	1.24	<0.001	1.12	1.39
	2	1.47	<0.001	1.40	1.54
	4	1.12	<0.001	1.08	1.17
	5	0.92	0.101	0.84	1.01
	6	1.70	<0.001	1.61	1.80

**Fig. 4:**
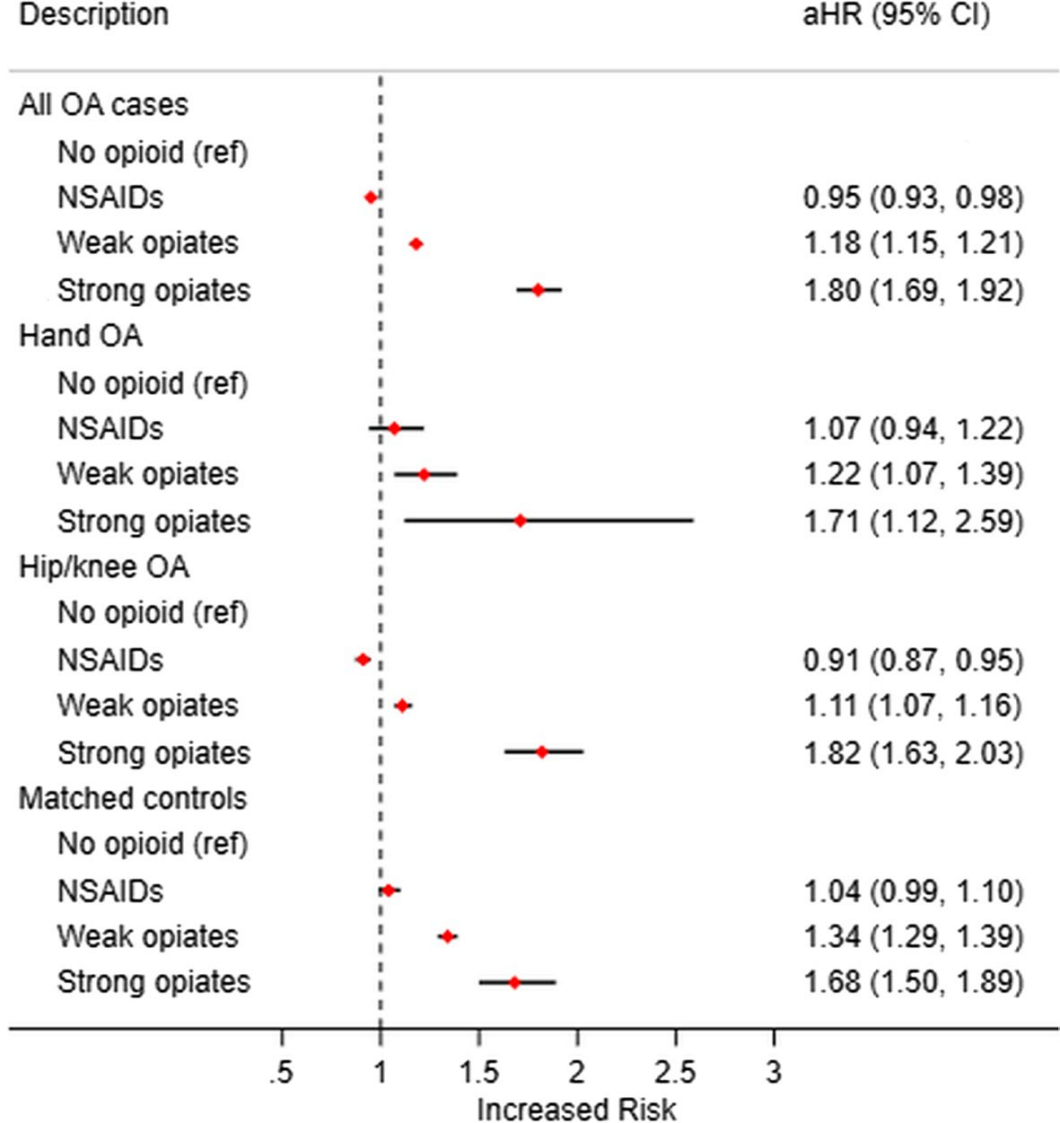
The effect of opioid use on mortality stratified by OA type and in the control population. Models were adjusted for comorbidity phenotypes, age, sex, BMI categories, deprivation quintiles, smoking status, and ethnicity

The impact of opioid use in the first 12 months of diagnosis on the hazards of mortality is shown in Fig. 4. Compared to no opioid use, the adjusted hazards of mortality were significantly greater for weak opioid and strong opioid users across all four groups. There was however no evidence of association between NSAID use and altered risk of mortality. In the matched control population, similar patterns were observed as the overall OA cases, but the effect sizes were reduced for the strong opiates (HR 1.68 (95% CI 1.50–1.89)) and slightly increased for weak opiates (HR 1.34 (95% CI 1.29–1.39)).

## Discussion

### Summary of results

Our analysis shows that OA is associated with multimorbidity, that distinct multimorbidity clusters exist in OA, and that there are differences in these clusters between hand and hip-knee subtypes. Patients across all subtypes of OA were predominantly female. A high prevalence of hypertension and metabolic disease was observed in all subtypes of OA as well as the majority of comorbidity clusters. All clusters, apart from the high morbidity burden cluster with higher proportions of the metabolic syndrome (cardiovascular, metabolic, and hypertensive disease) and renal disease (cluster 6), were associated with better survival in hand in comparison to hip-knee OA (Fig. 2). Higher mortality was observed in those with OA taking opioids, compared to those not, across all OA cases. NSAID use was not associated with higher mortality across all OA cases; however, slightly increased mortality was observed in hand OA in comparison to hip OA.

### Results in the context of the published literature

Our data are consistent with findings from cross-sectional epidemiological research [21] and the Global Burden of Disease 2010 Study [22] — OA has a predominantly female prevalence, particularly hand OA. The association between the metabolic syndrome and hip-knee OA is well documented [23, 24]; however, our data demonstrate that in the context of multimorbidity clusters the metabolic syndrome is highly prevalent across several clusters in both hip-knee and hand OA — in particular cluster 6.

Most patients with OA had at least one comorbid condition, supporting the recent increased scientific enquiry into OA and comorbidity. A systematic review reported that the commonest comorbidities associated with OA are stroke, peptic ulcer, and metabolic syndrome [7]. Similar findings were reported from a large cross-sectional study based in primary care [25]. Our data supports this, with a high prevalence of metabolic disease and hypertension seen across multiple clusters and with a higher prevalence in OA in comparison to the general population (Supplementary table S2). However, identification and analysis of multiple multimorbidity clusters in OA in a sample of our size have not been reported before.

The association between OA and mortality has been examined before but no firm conclusions drawn due to low-quality evidence and heterogeneity between studies [26, 27]. Our data set is one of the largest to date. As far as we are aware, this analysis is the first to demonstrate differences in mortality between OA subtypes and association of analgesic use with mortality within a cohort of individuals with OA. Hip/knee patients had worse mortality outcomes in comparison to those with hand OA. No adverse effect of NSAIDs was found, demonstrating that NSAIDs can provide a safe pharmacological management option in OA. This is reassuring given their potential for gastro-intestinal and cardiovascular toxicity [28–30]. We recognise that individuals without OA may be taking non-prescription NSAIDs at a greater rate than those with OA, which may account for the increased hazard of mortality seen in the controls with NSAID use.

Increased mortality risk with opioids in non-cancer pain is well documented [31, 32], though little is known about mortality risk with opioids in OA. A large cohort study of patients with OA identified a higher risk of mortality with opioids; however, these results may be prone to confounding by indication [13]. We demonstrate worse mortality outcomes for those taking weak and strong opioids across all OA and control populations. We also found slightly lower hazard ratios for mortality for weak opioid subgroups in OA, in comparison to controls. It is difficult to draw firm conclusions as the indications for opioids in the control group are likely to be very heterogeneous.

Results were adjusted for social deprivation, BMI, and smoking status. However, other reasons beyond the scope of our data that could explain mortality differences include patterns of healthcare usage, physical activity, and diet [33, 34].

### Internal validity

A major strength of our study is that it has a large sample size and data on 30 different comorbidities from a primary care database rather than self-reported data, which tends to overestimate certain disease prevalence [35], making this one of the largest OA data sets studied. The prevalence of major chronic conditions in IMRD-UK, adjusted for patient demographics, is similar to national estimates and patients in this database are also broadly representative of the UK general population [14]. A major advantage of the clustering method used is that LCA utilises a robust model-based probabilistic approach that does not rely on an arbitrary choice of metrics, and it is generally regarded as superior to traditional cluster analysis [18]. However, LCA is exploratory by nature and the resulting phenotypes are determined by the medical conditions included in the analysis. The entropy obtained for our models was also lower than the recommended 0.8 indicating a low degree of separation between the classes.

Another strength of our analysis is that OA is relatively straight-forward to diagnose in clinical practice, enabling greater reliability for coding. Conversely, the main limitation is that coding for OA is binary — Read Codes for OA do not account for undiagnosed disease or disease severity [36]. We acknowledge a limitation of our analysis is not having information on over-the-counter analgesics, such as low-dose ibuprofen and co-codamol (which contains codeine) and only include analgesics prescribed by general practitioners.

### Clinical significance

#### Multimorbidity clusters in osteoarthritis

Our analysis confirms that OA does not exist in isolation to other diseases and patients with OA are likely to have multimorbidity. The traditional paradigm of OA as a disease of joint ‘wear and tear’, leading to reduced mobility and sequelae of obesity, and metabolic syndrome, does explain to an extent the presence of multimorbidity in OA [23]. A newer paradigm proposes that as the risk of OA and a broad range of comorbidities, such as cardiovascular disease, increases with advancing age, they share an underlying pathophysiology based on biological ageing processes [37, 38]. These ageing processes, termed the hallmarks of ageing, include cell senescence, a state of irreversible cell-cycle arrest, and altered proteostasis seen in multiple age-related diseases including OA [38–41]. The reality is likely to be somewhere in between these two paradigms, with our analysis providing the following insights: (i) observed differences in multimorbidity clustering and disease prevalence between hand and hip-knee OA subtypes support differences in their pathophysiology; (ii) the higher prevalence of comorbidities in OA with a metabolic or inflammatory pathogenesis, such as type 2 diabetes mellitus, cardiovascular disease, and hypertension, compared to controls support growing evidence that OA is not just a biomechanical disease but a disease with a metabolic/inflammatory component [42, 43].

We demonstrated that patients with OA are more likely to have comorbid disease than the general population — particularly the metabolic syndrome. We therefore recommend that pro-active case finding of these diseases is integrated within the management of OA — for example, monitoring of HBA1c, cholesterol, and blood pressure. The management of multiple body systems means that patients with OA are likely to benefit from the input of generalists, such as general practitioners and geriatricians.

Interestingly, the high burden cluster with a higher prevalence of circulatory/metabolic/hypertensive disease and the highest prevalence of renal disease (cluster 6) had twice the hazard of mortality in hand OA, in comparison to the mortality observed in hip/knee OA. Management of these comorbidities in clinical practice is often intertwined: diabetes causes nephropathy, both of which are risk factors for cardiovascular disease [44]; hypertension is more prevalent in diabetics and increases cardiovascular disease risk [45]; the interactions between renal and cardiovascular disease are complex and interdependent [46]. It is not possible to establish causation from our analysis; furthermore, the interconnected management of these comorbidities means it is difficult to identify a dominant comorbidity within this cluster accounting for increased mortality in hand OA.

#### Analgesic use and mortality in osteoarthritis

Our analysis of this OA cohort demonstrates an increased risk of mortality with opioid use, particularly strong opioids in hip-knee OA. This may be explained by the higher prevalence of renal disease in hip-knee in comparison to hand OA and control groups — excretion of opioids is impaired in renal disease [47, 48]. Opioid prescription is also a marker of severe OA [49, 50].

Weak opioid and NSAID usage in hand OA was also associated with a slightly higher hazard of mortality in comparison to hip/knee OA. Although it is acknowledged subchondral bone and structural features do not necessarily correlate with pain severity [51], it could be differences in the quality or character of pain in hand OA versus hip/knee OA [52] account for greater dependency on NSAIDs/weak opioids in hand OA. In addition, differences in cluster composition, as demonstrated by the high burden cluster with metabolic syndrome and renal disease (cluster 6), may contribute to increased mortality with weak opioids and NSAIDs in hand OA — non-aspirin NSAIDs are associated with increased cardiovascular events [29, 53] and can precipitate acute kidney injury [54].

The clinical implications are that opioid-sparing options such as physiotherapy, steroid injections, and surgery should be used early and in preference to opioids, to prevent disease progression and opioid prescription. If surgery is required, early identification of multimorbidity with input from perioperative medicine services is recommended given the complexities of managing multimorbidity in the perioperative period. If opioids cannot be avoided, they should be used with caution and for the shortest duration possible. These points are particularly important for patients with hip-knee OA and those belonging to higher morbidity burden clusters to improve prognosis.

Results from this cohort suggest that a different approach to OA management may be required in hand versus hip-knee OA, particularly in the presence of metabolic syndrome and renal disease. DMARDs have not demonstrated clinical improvements in osteoarthritis pain, suggesting inflammation may not be the main contributor to OA pain [55]. It is now recognised that hallmarks of ageing contribute to abnormal cartilage homeostasis [41, 56, 57]. Increasing evidence demonstrates that these hallmarks can be ameliorated through targeted pharmaceuticals — in mouse models, intra-articular injection of rapamycin (an autophagy-modulator) and a senolytic agent (a drug that clears senescent cells) delayed cartilage degeneration and attenuated development of OA pain [58, 59], although a 12-week clinical trial of the senolytic UBX0101 failed to demonstrate symptomatic benefit [60]. Modulation of superficial zone integrity with intra-articular injection of lubricin has also demonstrated pain reduction [61]. This targeted approach could offer a potential novel intervention to reduce the need for NSAIDs and opioid usage; however, further clinical trials are required before introduction into clinical practice.

## Conclusion

Our analysis demonstrates the presence of distinct multimorbidity clusters in OA and matched control populations. There is a high prevalence of metabolic disease and hypertension across multiple clusters and a higher prevalence of these diseases in hip-knee OA compared to hand OA. Survival is higher in hand OA compared to hip-knee OA in all multimorbidity clusters apart from the high morbidity burden cluster. Increased risk of mortality was observed for weak and strong opioids across all OA and control populations, though no detrimental effect of NSAIDs was seen. Recommendations for clinical practice from our analysis are (1) pro-active case finding of comorbid disease is integrated within the management of OA, (2) that generalists are involved in the management of patients with OA and multimorbidity, and (3) opioid-sparing treatment options are used early and in preference to opioids particularly for patients with hip-knee OA and/or belonging higher morbidity burden clusters to improve prognosis. Further research is required to determine whether the multimorbidity clusters identified in our analysis are found in the general population, an understanding of trajectories towards the clusters particularly the temporal relationship between OA and associated comorbidities, and the pathophysiological processes underlying OA subtypes.

## Supplementary Information

**Additional file 1: Supplementary methods**. **Supplementary table S1**: Read codes used to identify diagnoses of OA and their subtypes by region. **Supplementary table S2**: Prevalence of comorbid conditions by type of OA. **Supplementary table S3**: Grouping of individual diseases into comorbidity groups. **Supplementary table S4**: Model fit statistics and classification quality.

## Data Availability

Anonymized patient-level data and statistical analysis codes can be made available on reasonable request. Requests should be made to author K.N. (k.nirantharan@bham.ac.uk).

## Abbreviations

OA: Osteoarthritis
LCA: Latent class analysis
IMRD-UK: IQVIA Medical Research Data UK
NSAIDs: Non-steroidal anti-inflammatory drugs
HRs: Hazard ratios
CI: Confidence interval
BMI: Body mass index
IQR: Interquartile range
