# Supplementary material for: Comorbidity phenotypes and risk of mortality in patients with osteoarthritis in the UK: a latent class analysis
Source: Arthritis Res Ther. 2022 Oct 13;24:231. doi: 10.1186/s13075-022-02909-4 (PMC9559033; doi:10.1186/s13075-022-02909-4)
Supplement: Supplementary file 1 — Additional file 1: Supplementary methods. Supplementary table S1: Read codes used to identify diagnoses of OA and their subtypes by region. Supplementary table S2: Prevalence of comorbid conditions by type of OA. Supplementary table S3: Grouping of individual diseases into comorbidity groups. Supplementary table S4: Model fit statistics and classification quality. [file 13075_2022_2909_MOESM1_ESM.docx]

# SUPPLEMENTARY MATERIAL

**Supplementary methods**

**Latent class analysis (LCA)**

LCA is a statistical method used for finding unobservable, or latent, subgroups within a population based on the similarity of patterns across a given data. In this method, posterior probabilities are assigned to each individual based on the estimated model parameters and their observed scores. This enables each individual to be allocated to the appropriate latent class based on their probability of membership. Following this, the risk of adverse outcomes (in this case mortality) by cluster could be estimated.

Due to the large size of the population in this analysis, the LCA Stata Plugin was used to undertake the LCA. This Plugin can be used to estimate latent classes that are measured by categorical indicators (Lanza et al. 2018). The standard method in Stata, generalised structural equation modelling (gsem) command, was unable to handle the data in this study. Models with class (cluster) size of 2-8 were run in each of the groups, and the optimal number of clusters in the underlying data was selected based on the following criteria:

A – Model fit statistics (Supplementary table S4): while the eight-class solution was preferred in most criteria of minimising AIC, adjusted BIC, and maximising the log-likelihood, there was not a lot of added benefit for these criteria above the six-class solution as shown in the log-likelihood plot below.


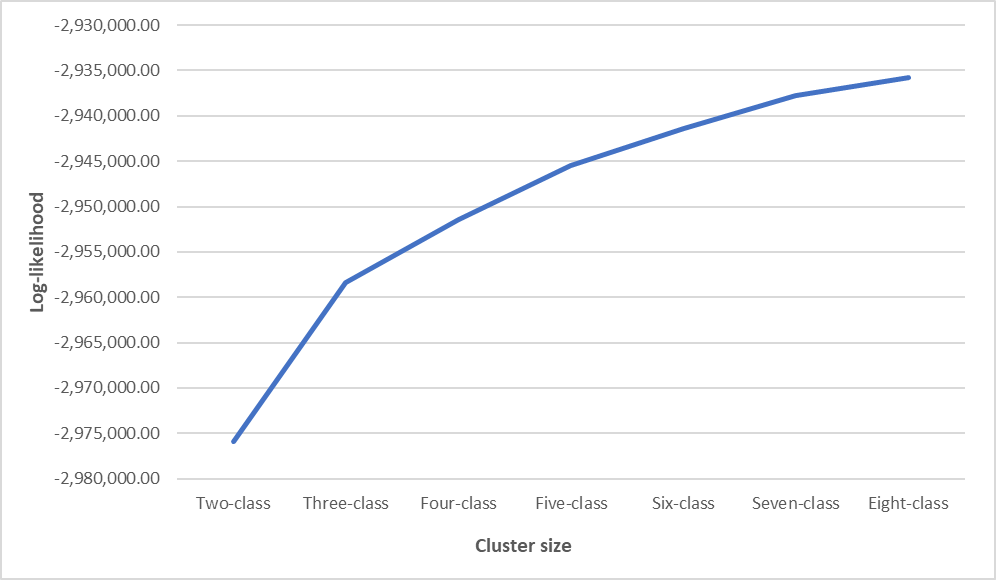


B - No small clusters: The rule of thumb in LCA clustering is that there should ideally be no cluster size below 5% of the study population. With seven or more clusters, at least two of the clusters were below 5%. The choice of six clusters meant that there was only one cluster (cluster 1) in each of the LCA conducted that made up below 5% of the population.

C - Domain-usefulness: In terms of clinical interpretability and meaningfulness, the six clusters were found to be more distinct and clinically interpretable than the alternative clusters considered across the sub-groups.

**References**

Lanza, S. T., Dziak, J. J., Huang, L., Wagner, A. T. , & Collins, L. M. (2018). LCA Stata plugin users' guide (Version 1.2.1). University Park: The Methodology Center, Penn State. Available from methodology.psu.edu.

**Supplementary tables**

Supplementary table S1: Read codes used to identify diagnoses of OA and their subtypes by region.

| **Read code** | **Description** | **OA type** |
| --- | --- | --- |
| N05z712 | Foot osteoarthritis NOS | Site unspecified |
| N052000 | Localised, secondary osteoarthritis of unspecified site | Site unspecified |
| N05z711 | Ankle osteoarthritis NOS | Site unspecified |
| N054300 | Oligoarticular osteoarthritis, unspecified, of forearm | Site unspecified |
| N05z713 | Toe osteoarthritis NOS | Site unspecified |
| N050100 | Generalised osteoarthritis of the hand | Hand |
| N052400 | Localised, secondary osteoarthritis of the hand | Site unspecified |
| N054700 | Oligoarticular osteoarthritis, unspecified, of ankle/foot | Site unspecified |
| N053611 | Patellofemoral osteoarthritis | Site unspecified |
| N050500 | Secondary multiple arthrosis | Site unspecified |
| N052800 | Localised, secondary osteoarthritis of other specified site | Site unspecified |
| N050z00 | Generalised osteoarthritis NOS | Site unspecified |
| N05zG00 | Osteoarthritis NOS, of PIP joint of finger | Hand |
| N051C00 | Primary arthrosis of first carpometacarpal joints, bilateral | Hand |
| N05zK00 | Osteoarthritis NOS, of sacro-iliac joint | Site unspecified |
| N051G00 | Osteoarthritis of spinal facet joint | Site unspecified |
| N05..00 | Osteoarthritis and allied disorders | Site unspecified |
| N05z311 | Wrist osteoarthritis NOS | Site unspecified |
| N05zS00 | Osteoarthritis NOS, of 1st MTP joint | Site unspecified |
| N051.00 | Localised, primary osteoarthritis | Site unspecified |
| N053100 | Localised osteoarthritis, unspecified, of shoulder region | Site unspecified |
| N05z700 | Osteoarthritis NOS, of ankle and foot | Site unspecified |
| N050111 | Heberdens' nodes | Hand |
| N051200 | Localised, primary osteoarthritis of the upper arm | Site unspecified |
| N053500 | Localised osteoarthritis, unspecified, pelvic region/thigh | Site unspecified |
| N050112 | Bouchards' nodes | Hand |
| N051600 | Localised, primary osteoarthritis of the lower leg | Site unspecified |
| N053900 | Arthrosis of first carpometacarpal joint, unspecified | Hand |
| N053z00 | Localised osteoarthritis, unspecified, NOS | Site unspecified |
| N05zC00 | Osteoarthritis NOS, of elbow | Site unspecified |
| N05..11 | Osteoarthritis | Site unspecified |
| N05z300 | Osteoarthritis NOS, of the forearm | Site unspecified |
| N050000 | Generalised osteoarthritis of unspecified site | Site unspecified |
| N053511 | Otto's pelvis | Site unspecified |
| N054600 | Oligoarticular osteoarthritis, unspecified, of lower leg | Site unspecified |
| N052300 | Localised, secondary osteoarthritis of the forearm | Site unspecified |
| N050400 | Primary generalized osteoarthrosis | Site unspecified |
| N053512 | Hip osteoarthitis NOS | Hip/knee |
| N052700 | Localised, secondary osteoarthritis of the ankle and foot | Site unspecified |
| N05zJ00 | Osteoarthritis NOS, of hip | Hip/knee |
| N051B00 | Primary gonarthrosis, bilateral | Site unspecified |
| N05zN00 | Osteoarthritis NOS, of ankle | Site unspecified |
| N05z.11 | Joint degeneration | Site unspecified |
| N051F00 | Localised, primary osteoarthritis of elbow | Site unspecified |
| N05zR00 | Osteoarthritis NOS, of other tarsal joint | Site unspecified |
| N05z211 | Elbow osteoarthritis NOS | Site unspecified |
| N054.00 | Oligoarticular osteoarthritis, unspecified | Site unspecified |
| N05z611 | Knee osteoarthritis NOS | Hip/knee |
| N053000 | Localised osteoarthritis, unspecified, of unspecified site | Site unspecified |
| N051100 | Localised, primary osteoarthritis of the shoulder region | Site unspecified |
| N053400 | Localised osteoarthritis, unspecified, of the hand | Hand |
| N051500 | Localised, primary osteoarthritis of the pelvic region/thigh | Site unspecified |
| N053800 | Localised osteoarthritis, unspecified, of other spec site | Site unspecified |
| N05zB00 | Osteoarthritis NOS, of acromioclavicular joint | Site unspecified |
| N051900 | Primary coxarthrosis, bilateral | Site unspecified |
| N051z00 | Localised, primary osteoarthritis NOS | Site unspecified |
| N05zF00 | Osteoarthritis NOS, of MCP joint | Hand |
| N052C00 | Post-traumatic gonarthrosis, unilateral | Site unspecified |
| N05z.00 | Osteoarthritis NOS | Site unspecified |
| N05z200 | Osteoarthritis NOS, of the upper arm | Site unspecified |
| N05z600 | Osteoarthritis NOS, of the lower leg | Site unspecified |
| N054100 | Oligoarticular osteoarthritis, unspecified, of shoulder | Site unspecified |
| N052200 | Localised, secondary osteoarthritis of the upper arm | Site unspecified |
| N054500 | Oligoarticular osteoarthritis, unspecified, of pelvis/thigh | Site unspecified |
| N050300 | Bouchard's nodes with arthropathy | Hand |
| N052600 | Localised, secondary osteoarthritis of the lower leg | Site unspecified |
| N050700 | Heberden's nodes with arthropathy | Hand |
| N054900 | Oligoarticular osteoarthritis, unspecified, multiple sites | Site unspecified |
| N054z00 | Osteoarthritis of more than one site, unspecified, NOS | Site unspecified |
| N051A00 | Coxarthrosis resulting from dysplasia, bilateral | Site unspecified |
| N05zM00 | Osteoarthritis NOS, of tibio-fibular joint | Site unspecified |
| N051E00 | Localised, primary osteoarthritis of toe | Site unspecified |
| N05zQ00 | Osteoarthritis NOS, of talonavicular joint | Site unspecified |
| N05zU00 | Osteoarthritis NOS, of IP joint of toe | Site unspecified |
| N052.00 | Localised, secondary osteoarthritis | Site unspecified |
| N05z511 | Hip osteoarthritis NOS | Hip/knee |
| N05zz00 | Osteoarthritis NOS | Site unspecified |
| N051000 | Localised, primary osteoarthritis of unspecified site | Site unspecified |
| N053300 | Localised osteoarthritis, unspecified, of the forearm | Site unspecified |
| N05z900 | Osteoarthritis NOS, of shoulder | Site unspecified |
| N051400 | Localised, primary osteoarthritis of the hand | Hand |
| N053700 | Localised osteoarthritis, unspecified, of the ankle and foot | Site unspecified |
| N051800 | Localised, primary osteoarthritis of other specified site | Site unspecified |
| N05zA00 | Osteoarthritis NOS, of sternoclavicular joint | Site unspecified |
| N05zE00 | Osteoarthritis NOS, of wrist | Site unspecified |
| N052B00 | Post-traumatic arthrosis of first carpometacarpal jt bilat | Site unspecified |
| N05z100 | Osteoarthritis NOS, of shoulder region | Site unspecified |
| N05z500 | Osteoarthritis NOS, pelvic region/thigh | Site unspecified |
| N050.00 | Generalised osteoarthritis - OA | Site unspecified |
| N054400 | Oligoarticular osteoarthritis, unspecified, of hand | Hand |
| N052100 | Localised, secondary osteoarthritis of the shoulder region | Site unspecified |
| N050200 | Generalised osteoarthritis of multiple sites | Site unspecified |
| N054800 | Oligoarticular osteoarthritis, unspecified, other spec sites | Site unspecified |
| N052500 | Localised, secondary osteoarthritis of pelvic region/thigh | Site unspecified |
| N050600 | Erosive osteoarthrosis | Site unspecified |
| N052z00 | Localised, secondary osteoarthritis NOS | Site unspecified |
| N052900 | Post-traumatic coxarthrosis, bilateral | Site unspecified |
| N05zH00 | Osteoarthritis NOS, of DIP joint of finger | Hand |
| N051D00 | Localised, primary osteoarthritis of the wrist | Site unspecified |
| N05zL00 | Osteoarthritis NOS, of knee | Hip/knee |
| N05zP00 | Osteoarthritis NOS, of subtalar joint | Site unspecified |
| N05z411 | Finger osteoarthritis NOS | Hand |
| N054000 | Oligoarticular osteoarthritis, unspec, of unspecified sites | Site unspecified |
| N05z412 | Thumb osteoarthritis NOS | Hand |
| N05zT00 | Osteoarthritis NOS, of lesser MTP joint | Site unspecified |
| N05z800 | Osteoarthritis NOS, other specified site | Site unspecified |
| N053200 | Localised osteoarthritis, unspecified, of the upper arm | Site unspecified |
| N051300 | Localised, primary osteoarthritis of the forearm | Site unspecified |
| N053600 | Localised osteoarthritis, unspecified, of the lower leg | Site unspecified |
| N051700 | Localised, primary osteoarthritis of the ankle and foot | Site unspecified |
| N05zD00 | Osteoarthritis NOS, of distal radio-ulnar joint | Site unspecified |
| N052A00 | Post-traumatic gonarthrosis, bilateral | Site unspecified |
| N05z000 | Osteoarthritis NOS, of unspecified site | Site unspecified |
| N05z400 | Osteoarthritis NOS, of the hand | Hand |
| N053.00 | Localised osteoarthritis, unspecified | Site unspecified |

Supplementary table S2: Prevalence of comorbid conditions by type of OA

| **Comorbidity** | **Hand OA** *26,005* | **%** | **Hip/knee OA** *137,507* | **%** | **OA Unknown** *254,817* | **%** | **All OA** *418,329* | **%** | **Controls** *243,170* | **%** |
| --- | --- | --- | --- | --- | --- | --- | --- | --- | --- | --- |
| Hypertension | 7,649 | 29.4 | 53,718 | 39.1 | 90,514 | 35.5 | 151,881 | 36.3 | 64,305 | 26.4 |
| Mental illness | 7,246 | 27.9 | 33,394 | 24.3 | 67,651 | 26.5 | 108,291 | 25.9 | 54,085 | 22.2 |
| Lung disease | 4,320 | 16.6 | 21,694 | 15.8 | 42,887 | 16.8 | 68,901 | 16.5 | 32,040 | 13.2 |
| Kidney disease (low eGFR) | 3,091 | 11.9 | 23,358 | 17.0 | 41,603 | 16.3 | 68,052 | 16.3 | 22,696 | 9.3 |
| Cardiovascular disease (CVD) | 2,916 | 11.2 | 21,779 | 15.8 | 39,554 | 15.5 | 64,249 | 15.4 | 25,093 | 10.3 |
| Gastrointestinal (GI) disease | 3,940 | 15.2 | 20,683 | 15.0 | 37,987 | 14.9 | 62,610 | 15.0 | 24,612 | 10.1 |
| Dyslipidaemia | 3,696 | 14.2 | 21,195 | 15.4 | 36,471 | 14.3 | 61,362 | 14.7 | 22,387 | 9.2 |
| Osteoporosis & fracture | 3,000 | 11.5 | 18,014 | 13.1 | 32,703 | 12.8 | 53,717 | 12.8 | 24,493 | 10.1 |
| Functional pain | 3,514 | 13.5 | 13,001 | 9.5 | 27,057 | 10.6 | 43,572 | 10.4 | 19,023 | 7.8 |
| Diabetes | 1,983 | 7.6 | 14,517 | 10.6 | 24,802 | 9.7 | 41,302 | 9.9 | 19,054 | 7.8 |
| Thyroid | 2,781 | 10.7 | 12,998 | 9.5 | 24,784 | 9.7 | 40,563 | 9.7 | 17,307 | 7.1 |
| Autoimmune/inflammatory disease | 2,414 | 9.3 | 12,097 | 8.8 | 24,378 | 9.6 | 38,889 | 9.3 | 19,128 | 7.9 |
| Cataract | 1,575 | 6.1 | 12,267 | 8.9 | 21,211 | 8.3 | 35,053 | 8.4 | 11,276 | 4.6 |
| Thromboembolism | 1,468 | 5.6 | 10,894 | 7.9 | 19,318 | 7.6 | 31,680 | 7.6 | 12,752 | 5.2 |
| Cancer | 1,635 | 6.3 | 9,798 | 7.1 | 16,438 | 6.5 | 27,871 | 6.7 | 13,760 | 5.7 |
| Urine incontinence | 1,996 | 7.7 | 8,522 | 6.2 | 17,069 | 6.7 | 27,587 | 6.6 | 10,031 | 4.1 |
| Gout | 1,119 | 4.3 | 8,021 | 5.8 | 13,950 | 5.5 | 23,090 | 5.5 | 7,869 | 3.2 |
| Arrhythmia | 1,128 | 4.3 | 8,202 | 6.0 | 13,573 | 5.3 | 22,903 | 5.5 | 8,787 | 3.6 |
| Anaemia | 1,325 | 5.1 | 6,839 | 5.0 | 13,678 | 5.4 | 21,842 | 5.2 | 9,756 | 4.0 |
| Erectile dysfunction | 1,066 | 4.1 | 8,269 | 6.0 | 12,033 | 4.7 | 21,368 | 5.1 | 9,937 | 4.1 |
| Benign prostatic hyperplasia (BPH) | 981 | 3.8 | 7,638 | 5.6 | 11,605 | 4.6 | 20,224 | 4.8 | 6,809 | 2.8 |
| Glaucoma | 578 | 2.2 | 4,141 | 3.0 | 7,349 | 2.9 | 12,068 | 2.9 | 4,772 | 2.0 |
| Heart valve disease | 441 | 1.7 | 2,874 | 2.1 | 4,769 | 1.9 | 8,084 | 1.9 | 3,149 | 1.3 |
| Macular degeneration | 281 | 1.1 | 2,259 | 1.6 | 3,851 | 1.5 | 6,391 | 1.5 | 1,893 | 0.8 |
| Retinal disease | 337 | 1.3 | 2,087 | 1.5 | 3,464 | 1.4 | 5,888 | 1.4 | 2,431 | 1.0 |
| Substance misuse | 253 | 1.0 | 1,308 | 1.0 | 2,687 | 1.1 | 4,248 | 1.0 | 2,298 | 0.9 |
| Chronic cystitis | 310 | 1.2 | 1,232 | 0.9 | 2,184 | 0.9 | 3,726 | 0.9 | 1,550 | 0.6 |
| Dementia | 128 | 0.5 | 1,274 | 0.9 | 2,304 | 0.9 | 3,706 | 0.9 | 1,948 | 0.8 |
| Chronic liver disease | 162 | 0.6 | 878 | 0.6 | 1,663 | 0.7 | 2,703 | 0.6 | 1,341 | 0.6 |
| Parkinson's disease | 54 | 0.2 | 657 | 0.5 | 1,119 | 0.4 | 1,830 | 0.4 | 850 | 0.3 |

Supplementary table S3: Grouping of individual diseases into comorbidity groups

| **Disease** | **Comorbidity group** |
| --- | --- |
| Arrhythmia | Circulatory |
| CVD |  |
| Heart valve disease |  |
| Thromboembolism |  |
| Diabetes | Metabolic |
| Dyslipidaemia |  |
| Thyroid disease |  |
| Gout |  |
| Chronic liver disease | Digestive |
| Gastrointestinal (GI) disease |  |
| Cataract | Eye |
| Glaucoma |  |
| Macular degeneration |  |
| Retinal disease |  |
| BPH | Genitourinary |
| Chronic cystitis |  |
| Erectile dysfunction |  |
| Urine incontinence |  |
| Substance misuse | Mental health |
| Mental illness |  |
| Parkinson's disease | Neurological |
| Dementia |  |
| Hypertension | Hypertension |
| Functional pain | Functional pain |
| Lung disease | Lung disease |
| Anaemia | Anaemia |
| Cancer | Cancer |
| Autoimmune/inflammatory | Autoimmune/inflammatory |
| Osteoporosis & fracture | Musculoskeletal |
| Kidney disease (low eGFR) | Kidney disease |

Supplementary table S4: Model fit statistics and classification quality

| LCA Model **Overall OA** | **Observations** | **Loglikelihood** | **AIC** | **Adjusted BIC** | **Entropy** |
| --- | --- | --- | --- | --- | --- |
| Two-class | 355,759 | -2,975,940.70 | 412,954.54 | 413,418.38 | **0.59** |
| Three-class | 355,759 | -2,958,414.30 | 377,963.83 | 378,663.39 | 0.53 |
| Four-class | 355,759 | -2,951,415.30 | 364,027.85 | 364,963.13 | **0.59** |
| Five-class | 355,759 | -2,945,431.80 | 352,122.80 | 353,293.81 | 0.54 |
| Six-class | 355,759 | -2,941,364.60 | 344,050.46 | 345,457.19 | 0.58 |
| Seven-class | 355,759 | -2,937,714.00 | 336,811.17 | 338,453.62 | 0.56 |
| Eight-class | 355,759 | **-2,935,725.60** | **332,896.31** | **334,774.49** | **0.59** |
| **Hand OA** |  |  |  |  |  |
| Two-class | 21,510 | -171,328.38 | 44,409.72 | 44,702.41 | 0.62 |
| Three-class | 21,510 | -170,398.27 | 42,611.50 | 43,052.94 | 0.56 |
| Four-class | 21,510 | -170,043.92 | 41,964.78 | 42,554.97 | 0.61 |
| Five-class | 21,510 | -169,751.97 | 41,442.88 | 42,181.82 | 0.58 |
| Six-class | 21,510 | -169,492.93 | 40,986.81 | 41,874.50 | **0.64** |
| Seven-class | 21,510 | -169,316.61 | 40,696.17 | 41,732.61 | 0.58 |
| Eight-class | 21,510 | **-169,193.31** | **40,511.56** | **41,696.75** | 0.63 |
| **Hip/Knee OA** |  |  |  |  |  |
| Two-class | 117,870 | -995,858.18 | 180,653.01 | 181,049.46 | 0.58 |
| Three-class | 117,870 | -990,209.56 | 169,417.76 | 170,015.70 | 0.51 |
| Four-class | 117,870 | -987,790.76 | 164,642.17 | 165,441.58 | 0.57 |
| Five-class | 117,870 | -985,828.40 | 160,779.44 | 161,780.33 | 0.55 |
| Six-class | 117,870 | -984,329.90 | 157,844.43 | 159,046.80 | **0.59** |
| Seven-class | 117,870 | -983,147.31 | 155,541.26 | 156,945.11 | 0.56 |
| Eight-class | 117,870 | **-982,448.75** | **154,206.15** | **155,811.48** | **0.59** |
| **Controls** |  |  |  |  |  |
| Two-class | 179,435 | -1,326,236.30 | 206,815.20 | 207,237.29 | 0.58 |
| Three-class | 179,435 | -1,317,467.30 | 189,339.19 | 189,975.79 | 0.58 |
| Four-class | 179,435 | -1,314,875.50 | 184,217.50 | 185,068.60 | 0.66 |
| Five-class | 179,435 | -1,312,546.70 | 179,621.96 | 180,687.56 | 0.67 |
| Six-class | 179,435 | -1,310,987.70 | 176,565.89 | 177,846.01 | **0.68** |
| Seven-class | 179,435 | -1,308,169.70 | 170,991.98 | 172,486.60 | 0.65 |
| Eight-class | 179,435 | **-1,306,569.60** | **167,853.66** | **169,562.78** | **0.69** |

Best model under each criterion highlighted in bold.
